# Combined Associations of Body Mass Index and Metabolic Health Status on Medical and Dental Care Days and Costs in Japanese Male Employees: A 4-Year Follow-Up Study

**DOI:** 10.2188/jea.JE20180268

**Published:** 2020-05-05

**Authors:** Kunihito Nishikawa, Masayuki Yamamoto

**Affiliations:** 1 Center of Medical Check-up, Shinko Hospital, Kobe, Japan; 2 Department of Environmental Epidemiology, Institute of Industrial Ecological Sciences, University of Occupational and Environmental Health, Kitakyushu, Japan; 3 Shin-Kobe Medical Examination Clinic, Shinko Hospital, Kobe, Japan

**Keywords:** medical costs, dental costs, oral health, obesity, metabolic syndrome

## Abstract

**Background:**

The combined associations of body mass index (BMI) levels and metabolic dysfunction with medical and dental care utilizations is unclear.

**Methods:**

A 4-year follow-up study was performed in 16,386 Japanese male employees (mean age 48.2 [standard deviation, 11.0] years) without a history of cardiovascular disease (CVD), cancer, or renal failure. They were classified into eight phenotypes based on four BMI levels (underweight, <18.5; normal weight, 18.5–24.9; overweight, 25.0–29.9; and obese, ≥30.0 kg/m^2^) and the presence or absence of ≥2 of 4 metabolic abnormalities: high blood pressure, high triglycerides, low high-density-lipoprotein cholesterol, and high blood sugar. Based on their health insurance claims data, we compared medical and dental care days and costs among the eight different BMI/metabolic phenotypes during 2010–2013.

**Results:**

The combinations of BMI levels and metabolic status were significantly associated with the adjusted mean and median medical outpatient days and costs and the median dental outpatient days and costs. The obese/unhealthy subjects had the highest medical outpatient days and costs, and the underweight/unhealthy subjects had the highest dental outpatient days and costs. The underweight/unhealthy subjects also had the highest medical inpatient days and hospitalization rates of CVD, and had higher medical costs compared with the obese/healthy subjects. The differences in median medical costs between healthy and unhealthy phenotypes were larger year by year across all BMI levels.

**Conclusions:**

Identification of obesity phenotypes using both BMI levels (including the underweight level) and metabolic status may more precisely predict healthcare days and costs compared with either BMI or metabolic status alone.

## INTRODUCTION

The World Health Organization (WHO) reported that approximately 36 million people die annually from non-communicable diseases, equaling 70% of all deaths in the world in 2008, and the annual number of deaths will increase to 55 million by 2030.^1^ Cardiovascular disease (CVD) is a leading cause (48% of non-communicable diseases), followed by cancers (21%), respiratory diseases (12%), and diabetes (3.5%).^1^ Dieleman et al predicted that the global healthcare costs will increase from 9.2 trillion United States dollars in 2014 to 24.2 trillion dollars in 2040.^2^ Effective prevention and treatment strategies against CVD and diabetes, commonly known as metabolic complications, are warranted to reduce the health and economic burdens.

Obesity is considered to be a fundamental risk factor for metabolic syndrome (MetS); however, the exact mechanisms underlying the development of MetS have not yet been established, and several institutions have suggested different criteria for MetS. In recent years, those who fit non-traditional obesity phenotypes (ie, metabolically unhealthy normal weight, MUNW; and metabolically healthy obesity, MHO) have been increasingly observed.^3^ The prevalence of these phenotypes is not low, and their increased risk of diseases and death should not be disregarded.^4^^–^^11^ These studies suggest that the risk of diabetes, renal disease, CVD, and death could be more precisely assessed using both body mass index (BMI) and metabolic status. BMI has a J-shaped relationship with medical hospital days^12^^,^^13^ and costs,^12^^,^^14^^,^^15^ and death.^16^^,^^17^ Studies on the combined associations of BMI levels and metabolic dysfunction on medical care needs are limited.^18^^,^^19^ Moreover, underweight (BMI <18.5 kg/m^2^) individuals are at substantial risk for mortality in Asia, Europe, and North America.^16^^,^^17^ However, in many studies, underweight individuals were excluded from the study subjects^4^^,^^5^^,^^19^ or included in the non-obese (<25.0 kg/m^2^) group together with normal-weight (18.5–24.9 kg/m^2^) individuals.^6^^–^^11^^,^^18^

Periodontal disease is a predominant oral disease worldwide,^20^ and its prevalence gradually increases with aging. This may contribute to the increasing dental costs in adults after reaching middle-age.^21^ Obesity and MetS are risk factors for periodontal disease, dental caries, and subsequent tooth loss.^22^^–^^25^ Poor oral health is likely to lead to systemic dysfunction, such as diabetes, MetS, and CVD.^26^^–^^29^ MetS and periodontal disease share common pathogenic backgrounds (eg, chronic inflammation and malnutrition) and have a bidirectional causal relationship. However, the relationship of different BMI and metabolic status combinations with dental care needs is unclear.

Therefore, we conducted a follow-up study to compare the medical and dental care utilizations during 2010–2013 among different BMI (including the underweight level) and metabolic health phenotypes in Japanese male employees, who have lower mean BMI than Caucasians.

## METHODS

### Study subjects

Our health screening institution conducts periodic health check-ups for the employees of the Kobe Steel Group, a major steel corporation in Japan, which has 13 production sites, 12 sales sites, and 212 associated subsidiary companies within the country. As all Japanese citizens are obliged to have medical insurance, all employees of this corporation are enrolled in the Kobe Steel Health Insurance Association (KSHIA). Of 20,574 employees who received an annually health examination including blood test after 10-hour fasting in 2010 (baseline year), 18,394 were enrolled KSHIA from 2010 through 2013. After excluding those with the following conditions: 1) a history of coronary heart disease (*n* = 128), stroke (*n* = 99), cancer (*n* = 94), or advanced renal failure (*n* = 32) in 2010 based on a self-completed questionnaire; 2) death (*n* = 75) or starting dialysis (*n* = 4) during 2010–2013 because of extremely high medical costs; 3) females (*n* = 1,639) because of the small percentage of female employees in this steel corporate group (9.3%); and 4) missing data (*n* = 104), 16,386 males (age: range 19–72, mean 48.2 [standard deviation {SD}], 11.0 years) were selected as subjects. We obtained all necessary data, excluding personal information (eg, names). Informed consent was obtained from all individual participants included in the study. This study was approved by the Ethics Committee of Shinko Hospital, Kobe, Japan (approval number: 1319).

### Data collection

A self-completed questionnaire was distributed to the subjects to clarify their disease history, smoking status, frequency and amount of alcohol (ethanol) consumption, exercise habits, and daily sleeping hours. These data were checked by physicians at the health check-up in 2010. The BMI was calculated by dividing the body weight (kg) by the square of the height (m). Blood pressure (BP) was measured in a sitting position after resting for at least 5 minutes. Blood samples were obtained after 10 hours of fasting. The total cholesterol, high-density lipoprotein cholesterol (HDL-C), low-density lipoprotein cholesterol (LDL-C), triglyceride (TG), blood sugar (BS), uric acid, and creatinine levels were analyzed. Urinary protein levels were evaluated using the dipstick method. The non-HDL-C was calculated as the total cholesterol minus HDL-C.

### Definition of obesity and metabolic abnormalities

According to BMI, the participants were divided into four categories: underweight (<18.5), normal weight (18.5–24.9), overweight (25.0–29.9), or obesity (≥30.0 kg/m^2^). These BMI categories correspond to the cut-off points proposed by the WHO. Using the Adult Treatment Panel-III (ATP-III) criteria,^30^ metabolic health status was evaluated based on whether subjects met 0–1 (metabolically “healthy”) or ≥2 (metabolically “unhealthy”) of the following four metabolic factors: 1) BP ≥130/85 mm Hg or use of hypertensive drugs, 2) TG ≥150 mg/dL or use of lipid-lowering drugs, 3) HDL-C <40 mg/dL, and 4) BS ≥100 mg/dL or use of medication for diabetes. Waist circumference was not used because of its collinearity with BMI. The subjects were categorized into eight groups (phenotypes) based on the four BMI levels and presence or absence of metabolically unhealthy status.

Underweight/unhealthy, normal weight/unhealthy (ie, metabolically unhealthy normal weight, MUNW), and obesity/healthy (ie, metabolically healthy obesity, MHO) phenotypes were regarded as non-traditional obesity phenotypes, exhibiting a discrepancy between BMI level and metabolic status.

### Follow-up and endpoints

Medical and dental fee receipts were sent monthly to the KSHIA by the hospitals that insured employees consulted, as shown in our previous study.^31^ We followed all receipts from January 2010 to December 2013. Receipts were divided into the following four categories: medical inpatient, medical outpatient, dental inpatient, and dental outpatient. For each subject, we calculated annual and 4-year cumulative numbers of medical and dental care days and costs during 2010–2013.

We also investigated medical and dental all-cause and CVD hospitalization events using the inpatient receipts on a monthly basis. Based on the International Classification of Diseases 10th revision (ICD-10), CVD was defined as diseases coded I00 to I99. If a code of I00 to I99 was present within the maximum four coded diagnoses on a medical inpatient receipt, the subject was considered to have been hospitalized due to CVD.

### Statistical analysis

Costs were expressed in Japanese yen (JPY) (1,000 JPY = 8.8 United States dollars or 7.8 euros, according to foreign exchange rates on Dec 6, 2018). We assessed the adjusted mean and median care days (days/person/year) and costs (JPY/person/year) for each phenotype. Not only adjusted mean values but median values were assessed because actual costs usually have a skewed distribution. Age, smoking status, frequency and amount of alcohol consumption, exercise habits, daily sleeping hours, and treatment for hypertension, dyslipidemia, or diabetes were defined as confounding factors. Differences in health care needs among the eight different BMI/metabolic phenotypes were evaluated using an analysis of co-variance, chi-square test, or median test. The differences were additionally evaluated in the subgroup defined as subjects without treatment for hypertension, dyslipidemia, or diabetes at the baseline. The annual rise (ΔJPY/person/year) in median medical costs during 2010–2013 for each phenotype was calculated by fitting a linear regression line: y = *a*x + *b* (y is the cost, x is the calendar year, and *b* is the intercept). All analyses were performed using SPSS version 23.0 (IBM Corp. Armonk, NY, USA). *P* < 0.05 was considered statistically significant.

## RESULTS

Table 1 shows the baseline characteristics according to BMI levels and metabolic health status. The prevalence of underweight, normal weight, overweight, and obesity was 4.4% (*n* = 718), 66.7% (*n* = 10,926), 24.2% (*n* = 3,970), and 4.7% (*n* = 772), respectively. The frequency of unhealthy metabolic status increased with increasing BMI level (7.5%, 22.5%, 47.6%, and 63.5% for underweight, normal weight, overweight, and obesity, respectively). The proportion of normal weight or underweight in unhealthy subjects was 51.4% (2,515/4,893). Significant differences among the eight phenotypes were observed in all items tested. The four unhealthy phenotypes, especially the underweight/unhealthy phenotype, were 1) older; 2) had higher rates of current smoking and heavy drinking; and 3) had poorer results for metabolic factors, uric acid, creatinine, and proteinuria, compared with the four corresponding healthy phenotypes.

**Table 1.** Baseline characteristics according to BMI levels and metabolic health status

	Underweight		Normal weight		Overweight		Obesity		*P* value^a^
	(BMI <18.5)		(BMI 18.5–24.9)		(BMI 25.0–29.9)		(BMI ≥30.0)		
			
	Healthy	Unhealthy	Healthy	Unhealthy	Healthy	Unhealthy	Healthy	Unhealthy	
	(*n* = 664)	(*n* = 54)	(*n* = 8,465)	(*n* = 2,461)	(*n* = 2,082)	(*n* = 1,888)	(*n* = 282)	(*n* = 490)	
Age, years	37.5 (13.0)	47.8 (14.0)	41.2 (12.7)	51.5 (10.3)	42.5 (11.5)	49.5 (10.2)	37.7 (10.8)	43.5 (10.8)	<0.001
Lifestyles									
Smoking status									<0.001
Never	39.9	18.5	44.5	38.1	46.3	38.7	42.2	45.7	
Past	10.8	18.5	16.6	18.2	17.1	19.7	19.5	15.9	
<20 cigarettes/day	38.4	38.9	29.8	29.5	26.6	27.0	26.6	26.3	
≥20 cigarettes/day	10.8	24.1	9.1	14.2	10.0	14.6	11.7	12.0	
Alcohol (ethanol) intake: frequency									<0.001
Never	25.9	31.5	16.3	15.3	16.4	17.2	22.3	26.7	
≤4 days/week	37.5	22.2	34.9	21.9	39.2	27.9	47.9	37.3	
5–6 days/week	15.4	11.1	24.9	29.0	24.6	28.9	17.0	22.7	
Every day	21.2	35.2	23.8	33.8	19.8	26.1	12.8	13.3	
Alcohol (ethanol) intake: amount									<0.001
None or ≤20 g/day	66.9	51.8	53.9	43.2	49.0	45.4	55.3	51.2	
21–40 g/day	22.3	20.4	31.7	35.5	33.0	32.8	28.0	27.6	
≥41 g/day	10.8	27.8	14.4	21.3	18.0	21.8	16.7	21.2	
Exercise habits									<0.001
<1 day/week	61.7	63.0	49	47.8	46.3	47.7	46.1	50.8	
Daily sleeping hours									<0.001
<6 hours/day	39.8	40.7	39.6	35.5	42.6	42.5	40.8	49.6	
Metabolic factors									
BMI, kg/m^2^	17.8 (0.7)	17.8 (0.6)	22.0 (1.6)	22.8 (1.5)	26.5 (1.2)	26.8 (1.3)	32.3 (2.7)	32.6 (3.0)	<0.001
Systolic BP, mm Hg	115 (13)	130 (14)	120 (13)	133 (14)	124 (12)	134 (13)	127 (12)	137 (14)	<0.001
Diastolic BP, mm Hg	69 (10)	78 (11)	73 (10)	82 (10)	77 (10)	84 (10)	78 (10)	85 (11)	<0.001
Triglycerides, mg/dL	73 (61)	151 (145)	92 (53)	186 (141)	114 (74)	203 (145)	118 (53)	198 (116)	<0.001
HDL-C, mg/dL	68 (16)	62 (14)	63 (15)	57 (15)	56 (11)	51 (12)	52 (9)	49 (11)	<0.001
LDL-C, mg/dL	99 (28)	105 (42)	115 (29)	125 (32)	127 (30)	130 (32)	128 (31)	133 (33)	
Non HDL-C, mg/dL	113 (30)	129 (44)	132 (33)	156 (35)	147 (33)	163 (35)	148 (35)	164 (36)	<0.001
Blood sugar, mg/dL	90 (12)	111 (47)	91 (12)	108 (30)	93 (12)	109 (29)	93 (13)	114 (41)	<0.001
Treated hypertension	1.8	14.8	4.3	24.4	7.3	29.4	4.6	31.2	<0.001
Treated dyslipidemia	0	7.4	0.5	12.9	0.8	14.3	0.7	13.9	<0.001
Treated diabetes	0.6	7.4	0.8	9.4	1.1	11.9	1.1	16.3	<0.001
Others									
Uric acid, mg/dL	5.3 (1.1)	5.4 (1.1)	5.7 (1.1)	6.0 (1.2)	6.1 (1.2)	6.3 (1.3)	6.5 (1.2)	6.5 (1.4)	<0.001
Creatinine, mg/dL	0.73 (0.11)	0.68 (0.12)	0.77 (0.11)	0.77 (0.13)	0.79 (0.11)	0.79 (0.14)	0.79 (0.12)	0.79 (0.15)	<0.001
Urinary protein (dipstick test)									<0.001
<1+	97.7	98.1	99.2	97.2	98.5	95.6	98.2	91.8	
1+	1.7	1.9	0.6	1.9	1.2	3.1	1.8	5.3	
≥2+	0.6	0	0.2	1.0	0.2	1.3	0	2.9	

BMI, body mass index; BP, blood pressure; HDL-C, high-density lipoprotein cholesterol; LDL-C, low-density lipoprotein cholesterol.

Values are expressed as mean (SD) or percentage (%).

^a^*P* values for differences among 8 phenotypes were adjusted for age, smoking status, frequency and amount of alcohol intake, exercise habits, daily sleeping hours, and treatment of hypertension, dyslipidemia, or diabetes.

During the follow-up period, 2,469 and 37 subjects had a medical and dental hospitalization event, respectively. For all subjects, the mean care days and costs were as follows: medical inpatient 0.63 (median, 0) and outpatient 7.7 (median, 4.3) days/person/year, and costs of 91,473 (median, 27,767) JPY/person/year; dental inpatient 0.004 (median, 0) and outpatient 2.9 (median, 1.8) days/person/year, and costs of 19,246 (median, 12,035) JPY/person/year, respectively.

Table 2 shows the 4-year cumulative medical care days and costs according to BMI levels and metabolic health status. Significant differences in the adjusted mean and median outpatient days and costs among the eight groups were observed. An unhealthy metabolic status increased medical outpatient days and costs regardless of BMI. The relationship between BMI and adjusted mean costs was J-shaped, with the nadir of the curve occurring at the normal-weight/healthy phenotype. However, the median value was the lowest at 15,996 JPY/person/year for the underweight/healthy phenotype, and it tended to increase with higher BMI levels and prevalent unhealthy metabolic status. The underweight/unhealthy subjects had higher adjusted mean and median medical costs than the obese/healthy subjects.

**Table 2.** 4-year cumulative medical care days and costs according to BMI levels and metabolic health status

	Underweight		Normal weight		Overweight		Obesity		*P* value^a^
	(BMI <18.5)		(BMI 18.5–24.9)		(BMI 25.0–29.9)		(BMI ≥30.0)		
			
	Healthy	Unhealthy	Healthy	Unhealthy	Healthy	Unhealthy	Healthy	Unhealthy	
	(*n* = 664)	(*n* = 54)	(*n* = 8,465)	(*n* = 2,461)	(*n* = 2,082)	(*n* = 1,888)	(*n* = 282)	(*n* = 490)	
Inpatient days, days/person/year									
Adjusted mean	0.65	1.49	0.61	0.64	0.56	0.65	0.86	0.77	0.670
(95% CI)	(0.34–0.95)	(0.43–2.55)	(0.53–0.70)	(0.48–0.81)	(0.39–0.73)	(0.47–0.84)	(0.40–1.33)	(0.42–1.13)	
Median	0	0	0	0	0	0	0	0	—
(interquartile range)	(0–0)	(0–0)	(0–0)	(0–0)	(0–0)	(0–0)	(0–0)	(0–0)	
Outpatient days, days/person/year									
Adjusted mean	7.7	8.9	7.4	7.9	7.6	8.7	9.2	9.7	<0.001
(95% CI)	(7.0–8.4)	(6.4–11.3)	(7.2–7.6)	(7.5–8.3)	(7.2–8.0)	(8.2–9.1)	(8.2–10.3)	(8.9–10.5)	
Median	2.8	4.0	3.3	7.8	4.0	9.0	4.0	9.3	<0.001
(interquartile range)	(1.0–6.8)	(1.0–14.6)	(1.5–7.8)	(2.8–14.8)	(1.5–9.0)	(3.5–15.5)	(1.5–9.0)	(4.3–15.8)	
Costs, 1,000 JPY/person/year^b^									
Adjusted mean	96	121	87	95	90	101	107	130	0.042
(95% CI)	(74–118)	(45–197)	(81–93)	(83–106)	(77–102)	(88–115)	(74–140)	(105–156)	
Median	16	29	20	56	26	68	23	75	<0.001
(interquartile range)	(6–45)	(8–104)	(8–55)	(17–134)	(10–70)	(22–145)	(9–83)	(27–178)	

BMI, body mass index; CI, confidence interval, JPY, Japanese yen.

^a^*P* values for differences among 8 phenotypes were adjusted for age, smoking status, frequency and amount of alcohol intake, exercise habits, daily sleeping hours, and treatment of hypertension, dyslipidemia, or diabetes.

^b^Cost unit is expressed as 1,000 JPY (1,000 JPY = 8.8 US dollars or 7.8 euros, according to foreign exchange rates on Dec 6, 2018).

Table 3 shows the 4-year cumulative dental care days and costs for different BMI and metabolic phenotypes. Inpatient days were very few. Regarding the median outpatient days and costs, significant differences were found among the eight phenotypes (*P* < 0.001). The median outpatient days and costs were higher if metabolic abnormalities were present regardless of BMI and highest in the underweight/unhealthy subjects.

**Table 3.** 4-year cumulative dental care days and costs for different BMI and metabolic phenotypes

	Underweight		Normal weight		Overweight		Obesity		*P* value^a^
	(BMI <18.5)		(BMI 18.5–24.9)		(BMI 25.0–29.9)		(BMI ≥30.0)		
			
	Healthy	Unhealthy	Healthy	Unhealthy	Healthy	Unhealthy	Healthy	Unhealthy	
	(*n* = 664)	(*n* = 54)	(*n* = 8,465)	(*n* = 2,461)	(*n* = 2,082)	(*n* = 1,888)	(*n* = 282)	(*n* = 490)	
Inpatient days, days/person/year									
Adjusted mean	0.003	0.000	0.004	0.003	0.006	0.000	0.001	0.019	0.203
(95% CI)	(−0.006 to 0.013)	(−0.032 to 0.032)	(0.001 to 0.007)	(−0.002 to 0.008)	(0.001 to 0.011)	(−0.006 to 0.006)	(−0.013 to 0.016)	(0.008 to 0.030)	
Median	0	0	0	0	0	0	0	0	0.938
(interquartile range)	(0–0)	(0–0)	(0–0)	(0–0)	(0–0)	(0–0)	(0–0)	(0–0)	
Outpatient days, days/person/year									
Adjusted mean	2.7	3.8	2.9	2.9	2.9	3.0	2.9	3.0	0.383
(95% CI)	(2.4–3.0)	(2.8–4.7)	(2.9–3.0)	(2.7–3.0)	(2.7–3.0)	(2.8–3.1)	(2.5–3.3)	(2.7–3.3)	
Median	1.3	2.6	1.8	2.3	1.8	2.3	1.5	1.8	<0.001
(interquartile range)	(0–3.3)	(0.4–7.3)	(0.3–4.0)	(0.3–5.0)	(0.1–4.0)	(0.3–5.0)	(0–4.3)	(0–4.5)	
Costs, 1,000 JPY/person/year^b^									
Adjusted mean	18	27	19	19	19	19	20	21	0.081
(95% CI)	(16–20)	(21–33)	(19–20)	(18–20)	(18–20)	(18–21)	(17–22)	(19–23)	
Median	9	17	11	15	11	15	10	13	<0.001
(interquartile range)	(0–23)	(2–48)	(1–26)	(2–33)	(0–27)	(2–34)	(0–28)	(0–30)	

BMI, body mass index; CI, confidence interval, JPY, Japanese yen.

^a^*P* values for differences among 8 phenotypes were adjusted for age, smoking status, frequency and amount of alcohol intake, exercise habits, daily sleeping hours, and treatment of hypertension, dyslipidemia, or diabetes.

^b^Cost unit is expressed as 1,000 JPY (1,000 JPY = 8.8 US dollars or 7.8 euros, according to foreign exchange rates on Dec 6, 2018).

Table 4 shows the 4-year cumulative medical and dental hospitalization rates for different BMI and metabolic phenotypes. The BMI and metabolic health combinations were associated with medical all-cause and CVD hospitalizations, but not with dental hospitalization. The rate of CVD hospitalization was highest in the underweight/unhealthy phenotype, and lowest in the normal-weight/healthy and underweight/healthy phenotypes.

**Table 4.** 4-year cumulative occurences of medical hospitalization for different BMI and metabolic phenotypes

	Underweight		Normal weight		Overweight		Obesity		*P* value^a^
	(BMI <18.5)		(BMI 18.5–24.9)		(BMI 25.0–29.9)		(BMI ≥30.0)		
			
	Healthy	Unhealthy	Healthy	Unhealthy	Healthy	Unhealthy	Healthy	Unhealthy	
	(*n* = 664)	(*n* = 54)	(*n* = 8,465)	(*n* = 2,461)	(*n* = 2,082)	(*n* = 1,888)	(*n* = 282)	(*n* = 490)	
Medical									
All-cause, %	14.8	21.8	14.1	15.3	15.8	16.2	20.0	20.5	<0.001
(95% CI)	(12.1–17.5)	(12.4–31.1)	(13.3–14.9)	(13.9–16.8)	(14.3–17.3)	(14.5–17.8)	(15.9–24.1)	(17.4–23.6)	
CVD, %	1.8	4.9	1.8	2.6	2.3	3.2	2.9	3.0	0.013
(95% CI)	(0.7–3.0)	(1.0–8.0)	(1.5–2.1)	(2.0–3.2)	(1.6–2.9)	(2.5–3.9)	(1.2–4.6)	(1.7–4.3)	
Ratio of CVD/All-cause, %	12.2	22.5	12.8	17.0	14.6	19.8	14.5	14.6	
Dental									
All-cause, %	0.3	0.0	0.2	0.2	0.3	0.2	0.0	0.2	0.925
	(0.0 to 0.7)	(−1.3 to 1.3)	(0.1 to 0.3)	(0.0 to 0.4)	(0.1 to 0.5)	(0.0 to 0.4)	(−0.5 to 0.6)	(−0.2 to 0.6)	

CVD, cardiovascular disease.

^a^*P* values for differences among 8 phenotypes were adjusted for age, smoking status, frequency and amount of alcohol intake, exercise habits, daily sleeping hours, and treatment of hypertension, dyslipidemia, or diabetes.

Additional evaluations with the subgroup (*n* = 13,825) after excluding 2,561 subjects with hypertension, dyslipidemia, or diabetes at the baseline indicated that the median medical outpatient days and costs were lower especially in the four unhealthy phenotypes than those in all subjects. However, similar results concerning the relationships of BMI/metabolic phenotypes and medical care utilizations were obtained from the subgroup as all subjects. Also, no remarkable differences were observed in dental care utilizations in each phenotype between the subgroup and all subjects.

**Figure 1.**
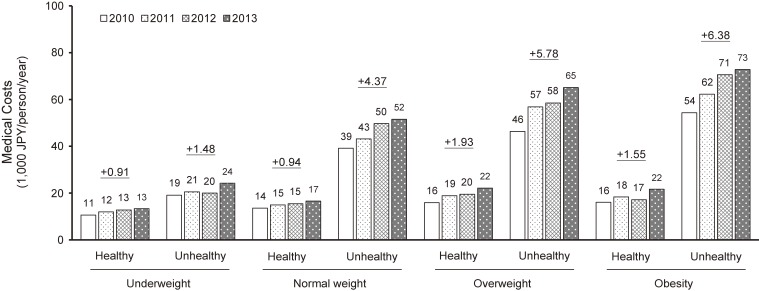
Median medical costs in each year and annual rise during 4 years based on BMI levels and metabolic health status *Note*. Cost unit is expressed as 1,000 JPY (1,000 JPY = 8.8 United States dollars or 7.8 euros, according to foreign exchange rates on Dec 6, 2018). Values with under lines expressed as the annual rise in medical costs for each phenotype (ΔJPY/person/year).

Figure 1 shows the median medical costs in each year and trends among different BMI and metabolic phenotypes. The annual rise in medical costs (ΔJPY/person/year) tended to increase with higher BMI levels and prevalent unhealthy metabolic status (the lowest rise at 906 JPY/person/year in the underweight/healthy phenotype; and highest at 6,379 JPY/person/year in obese/unhealthy phenotype). The differences in medical costs between healthy and unhealthy phenotypes were larger year by year across all BMI levels.

## DISCUSSION

Our major findings are as follows: 1) the different combinations of BMI levels and metabolic status were associated with the adjusted mean and median medical outpatient days and costs and median dental outpatient days and costs during the 4-year period; 2) the obese/unhealthy subjects had the highest medical outpatient days and costs, and the underweight/unhealthy subjects had the highest dental outpatient days and costs; 3) the underweight/unhealthy subjects had the highest medical inpatient days and hospitalization rates of all-cause and CVD, and more highly increased medical costs compared with the obese/healthy subjects. Our additional stratified analysis based on age of 45 years, nearly the mean age of all subjects, led to similar results to those mentioned above in both younger (*n* = 8,508) and older (*n* = 7,878) subjects. Essentially unchanged results were also observed in 13,825 subjects, even after excluding 2,561 subjects with treatment for hypertension, dyslipidemia, or diabetes at the baseline (data not shown). Therefore, identification of obesity phenotypes based on the combination of BMI (including underweight level) and metabolic status may be useful in selecting individuals with higher medical and dental care needs.

Obesity phenotypes have been defined in many ways in recent studies. Consequently, the prevalence of each phenotype varies widely across studies, affected by age distribution, gender, region, and, in particular, the criteria used for unhealthy metabolic status.^3^ Among our subjects, the prevalence of MHO, MUNW, and metabolically unhealthy obesity (MUO) was 1.7% (*n* = 282), 15.0% (*n* = 2,461), and 3.0% (*n* = 490), respectively. Similar to previous studies,^12^^,^^14^^,^^15^ the present study noted a J-shaped relationship between BMI and adjusted mean medical costs, with the nadir of the curve occurring at a BMI of 18.5–24.9 kg/m^2^ regardless of metabolic status. On the other hand, the median value was the lowest for the underweight/healthy phenotype, and it tended to increase with higher BMI levels and prevalent unhealthy metabolic status. Former studies have shown that the obese/unhealthy phenotype (ie, MUO) have the highest risk of diabetes, CKD, CVD, and death^5^^–^^9^ among all obesity phenotypes. These findings suggest that the MUO phenotype enhances the need for medical care services and accelerates costs compared with other obesity phenotypes.

Many studies have reported that MHO individuals may have a lower risk of health problems than MUNW and MUO individuals. However, some studies found that they had a higher risk of diabetes,^5^^,^^8^ CVD,^6^^,^^8^ and death^6^ than MUNW individuals in the long term. The present study revealed that overweight and obese healthy subjects had higher medical costs than normal-weight healthy subjects, consistent with previous studies.^18^^,^^19^ These findings suggest that identification of individuals at risk based only on metabolic status is insufficient. The MHO phenotype may be a transient status before the MUO phenotype. Therefore, a standardized approach to identify MHO individuals and initiate lifestyle interventions is needed to avoid progression to an unhealthy phenotype.

In the present study, the prevalence of the normal-weight/unhealthy (ie, MUNW) phenotype was not low (15.0%) in working-age Japanese males. This phenotype subjects had higher median medical costs and their annual rise compared with obese/healthy (ie, MHO) subjects. Some studies have indicated that MUNW individuals had an equivalent to or greater risk of CKD,^10^ CVD,^4^^,^^11^ and death^4^ than MUO individuals. These findings support that the clustering of metabolic factors is more strongly associated with medical costs than high BMI. The MUNW phenotype may have a large impact on medical costs, especially in Asians, who have a relatively lower prevalence of obesity than Caucasians. Non-obese individuals in a seemingly healthy condition are often considered to be at low risk and are managed accordingly. Therefore, the exclusion of this high-risk group from targets for intervention and follow-up is clinically and economically undesirable.

Underweight individuals are reported to have a higher risk of diseases and death than normal-weight individuals.^16^^,^^17^ Our results showed that the underweight/unhealthy subjects had the highest adjusted mean medical inpatient days and second highest adjusted mean medical costs. These results are partially explained by their highest rate of CVD hospitalization. They also had the highest dental outpatient days and costs. These findings may reflect the inverse causality of poor health leading to unintentional weight loss.^32^ However, the underweight/healthy subjects, not the normal-weight/healthy subjects, had the lowest rates of treatment for hypertension, dyslipidemia, and diabetes (Table 1), and the lowest median medical and dental outpatient days and costs. Metabolic dysfunction may contribute to increase medical and dental utilizations, especially at the underweight BMI level. These differences can be explained by the differences in the rates of current smoking (49.2% vs 63.0%), everyday alcohol intake (21.2% vs 35.2%), and excess alcohol intake of ≥41 g/day (10.8% vs 27.8%) between healthy and unhealthy underweight subjects (Table 1). Moreover, underweight unhealthy individuals may have a lower awareness of health-related behaviors and avoid visiting hospitals until diseases progress. These findings suggest that 1) the underweight BMI level (<18.5 kg/m^2^) should be separated from the non-obese level (<25.0 kg/m^2^) and 2) the presence or absence of metabolic dysfunction should be assessed when considering healthcare needs.

Periodontal disease is a chronic disease caused by local bacterial infection and is more frequent and severe in adulthood. However, dental care usage is reported to be less frequent even in developed countries. Of our subjects, 520 (3.2%) and 4,106 (25.1%) had no medical or dental visits over the 4-year period, respectively. Dental care access reflects not only oral conditions but age, sex, race, education, marital status, household income, and insurance coverage of dental services.^33^ Individuals without dental care use tend to be poor health status and lost permanent teeth.^33^^,^^34^ Periodontal disease is related with unfavorable lifestyles (eg, smoking), less frequent tooth-brushing, and less use of dental health services.^28^ The present study showed that the metabolically unhealthy subjects tended to have higher median dental costs than their healthy counterparts across all BMI levels, especially at the underweight level. Longitudinal studies have indicated that periodontal disease has a bidirectional relationship with MetS,^22^^,^^25^^–^^28^ suggesting that the control of MetS could reduce the risk of periodontal disease and vice versa. Therefore, more promotion of preventive dental services and education programs by dental professionals should be encouraged to reduce not only dental but medical health and economic burdens.

The present study had some limitations. First, the results of this study may differ from those obtained from different age ranges, sex, races, countries, health financing systems, and socioeconomic status. Second, annual changes in the adjusted mean medical costs and dental costs for each BMI/metabolic group varied too widely to assess trends during the follow-up, especially in smaller groups. A study with a larger sample size and a longer follow-up period is needed. Third, as receipts are essentially used to claim healthcare fees, if the patient has multiple diseases, these receipts do not show the details of fees and the number of days needed for each disease. Moreover, indirect economic burdens, such as lost work productivity costs and reduced quality of life, were not available. However, such receipts are valuable for accurate knowledge of healthcare days and costs.

In conclusion, improved identification of obesity phenotypes using both BMI levels (including the low range of <18.5 kg/m^2^) and metabolic health status may aid in predicting healthcare days and costs compared with BMI or metabolic status alone.
